# Differential effects of brain-derived neurotrophic factor and neurotrophin-3 on hindlimb function in paraplegic rats

**DOI:** 10.1111/j.1460-9568.2011.07950.x

**Published:** 2012-01

**Authors:** Vanessa S Boyce, Jihye Park, Fred H Gage, Lorne M Mendell

**Affiliations:** 1 Department of Neurobiology and Behavior, Life Sciences Building, Room 532, State University of New York at Stony BrookStony Brook, NY 11794-5230, USA; 2 Laboratory of Genetics, Salk InstituteLa Jolla, CA, USA

**Keywords:** c-Fos, locomotion, neurotrophin, plasticity, rheobase, spinal cord injury

## Abstract

We compared the effect of viral administration of brain-derived neurotrophic factor (BDNF) or neurotrophin 3 (NT-3) on locomotor recovery in adult rats with complete thoracic (T10) spinal cord transection injuries, in order to determine the effect of chronic neurotrophin expression on spinal plasticity. At the time of injury, BDNF, NT-3 or green fluorescent protein (GFP) (control) was delivered to the lesion via adeno-associated virus (AAV) constructs. AAV–BDNF was significantly more effective than AAV–NT-3 in eliciting locomotion. In fact, AAV–BDNF-treated rats displayed plantar, weight-supported hindlimb stepping on a stationary platform, that is, without the assistance of a moving treadmill and without step training. Rats receiving AAV–NT-3 or AAV–GFP were incapable of hindlimb stepping during this task, despite provision of balance support. AAV–NT-3 treatment did promote the recovery of treadmill-assisted stepping, but this required continuous perineal stimulation. In addition, AAV–BDNF-treated rats were sensitized to noxious heat, whereas AAV–NT-3-treated and AAV–GFP-treated rats were not. Notably, AAV–BDNF-treated rats also developed hindlimb spasticity, detracting from its potential clinical applicability via the current viral delivery method. Intracellular recording from triceps surae motoneurons revealed that AAV–BDNF significantly reduced motoneuron rheobase, suggesting that AAV–BDNF promoted the recovery of over-ground stepping by enhancing neuronal excitability. Elevated nuclear c-Fos expression in interneurons located in the L2 intermediate zone after AAV–BDNF treatment indicated increased activation of interneurons in the vicinity of the locomotor central pattern generator. AAV–NT-3 treatment reduced motoneuron excitability, with little change in c-Fos expression. These results support the potential for BDNF delivery at the lesion site to reorganize locomotor circuits.

## Introduction

Mechanical injury to the mammalian spinal cord initiates a cascade of biological processes that include loss of neurons via apoptotic cell death (Liu *et al.*, 1997) and production of inhibitory molecules that restrict axonal regeneration across the lesion (Schwab & Bartholdi, 1996). This cascade results in functional isolation of the spinal cord below the injury. Nonetheless, the spinal cord circuitry below the injury, particularly the circuitry responsible for locomotion, remains intact. This is evident from the ability to re-engage lumbar locomotor circuits via sensorimotor training in animal models of complete spinal injury to produce functional stepping (Lovely *et al.*, 1986; Barbeau & Rossignol, 1987; de Leon *et al.*, 1998a; Edgerton *et al.*, 2001; Leblond *et al.*, 2003), if performed in a timely manner (de Leon *et al.*, 1999; Norrie *et al.*, 2005).

Other than locomotor training, few broadly accepted clinical treatments for spinal cord injury (SCI) exist. Therefore, there is a need to identify interventions/molecules for acute application to the injured spinal cord to preserve and/or enhance the function of spared spinal circuitry. Our approach is to use neurotrophic factors, such as brain-derived neurotrophic factor (BDNF) or neurotrophin-3 (NT-3), to modify these spared spinal networks. These neurotrophins are excellent candidates for this purpose, as BDNF, NT-3 and their tropomyosin receptor kinase (Trk) receptors are widely expressed in the brain and spinal cord (Zhou *et al.*, 1993; Barbacid, 1994; Conner *et al.*, 1997), and are upregulated in the spinal cord of spinal-injured rats after training (Gomez-Pinilla *et al.*, 2001, 2002; Skup *et al.*, 2002; Ying *et al.*, 2003). They also promote regeneration (Schnell *et al.*, 1994; Liu *et al.*, 1999; Novikova *et al.*, 2002) and induce synaptic plasticity in the developing (Seebach *et al.*, 1999; Arvanian & Mendell, 2001; Cohen-Cory *et al.*, 2010) and adult (Lessmann, 1998; Baker-Herman *et al.*, 2004; Chen *et al.*, 2006) central nervous system. In addition, combination treatment of adult spinal cats with BDNF/NT-3-expressing fibroblasts restores treadmill locomotion and significantly enhances the locomotor recovery achieved with treadmill training (Boyce *et al.*, 2007). Combined Schwann cell transplants and adeno-associated virus (AAV)–BDNF/NT-3 treatment also improve stepping in adult spinal rats (Blits *et al.*, 2003).

Using the AAV delivery system to administer neurotrophins to the cord (Hermens & Verhaagen, 1998; Blits *et al.*, 2003; Hendriks *et al.*, 2004; Fortun *et al.*, 2009; Petruska *et al.*, 2010), we compared the effect of BDNF or NT-3 on locomotor recovery in adult rats after a complete SCI. Intraspinal AAV–BDNF or AAV–NT-3 administration immediately after complete spinal cord transection (Tx) resulted in very different locomotor outcomes. With balance support, AAV–BDNF promoted over-ground, weight-bearing stepping, which was unattainable with AAV–NT-3 treatment. We also established that AAV–BDNF and AAV–NT-3 had disparate effects on motoneuron physiology and on c-Fos expression in the lumbar cord, with AAV–BDNF being associated with increased excitability of spinal neurons vs. decreased excitability after AAV–NT-3 treatment. In so doing, we have identified a physiological signature of each neurotrophin that may predict locomotor recovery.

Preliminary accounts of this work have been presented elsewhere (Boyce *et al.*, 2010).

## Materials and methods

### Generation of AAV viral vectors

The methods used for generation of the viral vectors have been described previously (Kaspar *et al.*, 2002; Petruska *et al.*, 2010). In brief, the AAV constructs (pAAVsp and AAV; Salk Institute) contained a cytomegalovirus promoter, a synthetic intron flanked by splice donor/splice acceptor sites, and a multiple cloning site terminated by a β-globin polyA sequence. The transcript was flanked by AAV2 inverted terminal repeats. AAV (serotype 5) was generated with the pHelper plasmid and the capsid plasmid encoding Rep/Cap5.

Enhanced green fluorescent protein (240 amino acids) was cloned from the vector KS–green fluorescent protein (GFP) into AAV with *Eco*R1/*Xho*1. AAVsp–BDNF was cloned by use of the human BDNF coding sequence (CDS) (252 amino acids), including the secretory signal sequence. The human BDNF CDS was restriction digested from a modified pBluescript plasmid (SKps) by use of the *Sfi*1 and *Pme*1 sites, and then cloned into AAVsp with these same sites. AAVsp–NT-3 was cloned by use of the human NT-3 CDS (CDS 8538.1, 774 bp) corresponding to transcript variant 2 (NM_002527.4), which encodes the isoform 2 precursor protein (257 amino acids), including the secretory signal sequence. The human NT-3 CDS was restriction digested and cloned as stated above. Virus was purified via two rounds of CsCl density gradient centrifugation (Kaspar *et al.*, 2002; Petruska *et al.*, 2010), and titered by quantitative polymerase chain reaction (Virapur, CA, USA).

### SCI and application of neurotrophins

All procedures were approved by the Institutional Animal Care and Use Committee at the State University of New York at Stony Brook. These experiments were carried out with female Sprague–Dawley rats (5–6 months old, 300–310 g). Under isoflurane anesthesia (1.5–2% in O_2_) and with sterile procedures, the T10 spinal cord was exposed by dorsal laminectomy. The dura was opened with microscissors, and 0.1 mL of lidocaine (2% solution) was added dropwise to the surface of the cord to minimize the injury discharge that accompanies spinal Tx. The spinal cord was then completely transected at T10 with microscissors. As a result of Tx, the ends of the cord were separated by approximately 0.5–1 mm. With the surgical microscope and glass probes, this cavity was carefully inspected visually to confirm the absence of spared spinal tissue, and then filled with Gelfoam. Completeness of the lesion was also verified electrophysiologically and histologically (see Results).

Rats then received 2.5 × 10^10^ genomic copies of AAV5–BDNF (*n* = 6), AAV5–NT-3 (*n* = 6) or AAV5–GFP (*n* = 5) injected into the stump of the distal cord at the lesion site and into the lesion epicenter (Gelfoam) via a 1-mL Hamilton syringe. After the spinal cord had been covered with Durafilm or parafilm, muscle and skin layers were closed with 4.0 vicryl (Ethicon) and surgical staples, respectively. Isoflurane was discontinued, and the rats were transferred to their home cages, where Baytril (2.5 mg/kg), buprenorphine (0.1–0.5 mg/kg) and lactated Ringer’s solution were given via subcutaneous injection. The rats were housed individually and closely monitored during the recovery period. During this time, bladders were manually voided three times daily. Reflexive bladder voiding generally began within 2 weeks post-surgery.

### Behavioral assessments

Behavioral assessments were performed in all rats pre-injury to obtain baseline measures of locomotor ability and sensory responsiveness. Locomotor performance was evaluated with three stepping tasks: (i) walking over-ground across a 1.2-m stationary glass walkway enclosed by plexiglass panels, which were narrowed to provide balance support to the rat – under ambient light conditions, a lateral two-dimensional view of the locomotor movements was recorded with a tripod-mounted digital video camera, (at 30 frames per second), and this footage was later digitized for kinematic analysis; (ii) walking on the CatWalk apparatus in a darkened room for the collection and analysis of footprint data (Hamers *et al.*, 2006); and (iii) walking on a motorized treadmill (Lovely *et al.*, 1986; Boyce *et al.*, 2007; Alluin *et al.*, 2010). Sensory testing with the plantar heat test (Hargreaves *et al.*, 1988) was used to determine the response of the hindlimbs to noxious heat. Behavioral testing and analysis were conducted by individuals blinded to the treatment condition of each rat.

### Hargreaves test

The Hargreaves apparatus (Ugo Basile) was used to determine the latency of hindpaw withdrawal in response to a noxious heat stimulus. Testing was performed pre-injury and at 6 weeks post-Tx. Rats were placed in a behavioral chamber and allowed to acclimate for 20 min. A radiant heat source was then placed under each hindpaw and triggered to deliver a calibrated stimulus of 90 W/cm^2^. Hindpaw withdrawal stopped the automated timer, and the latency was recorded. This process was repeated for five trials, alternating between the left and right hindpaws of each rat. The highest and lowest values were discarded, and the remaining three latencies were averaged to give the mean withdrawal latency for each hindpaw.

### Footprint analysis

Footprint analysis was performed with the CatWalk device (Noldus) pre-Tx and at 2, 4 and 6 weeks post-Tx. These sessions were recorded in the dark, and the footfall patterns from each rat were captured by an infrared camera positioned under the glass walkway as the rats walked across it. Stepping behavior was recorded for three trials per rat at each time point. In order to obtain kinematic measures of this stepping, the CatWalk was modified such that the rats were filmed as they moved across the walkway (see Over-ground locomotion).

### Over-ground locomotion

Paint markers were placed on the skin over the ilium, femoral head, center of rotation of the knee, ankle, metatarsophalangeal (MTP) joint, and tip of the second digit of the hindlimb. Markers were also placed over the acromion process (shoulder), center of rotation of the elbow, metacarpophalangeal joint, and tip of the second digit of the forelimb. The rats were videotaped through a plexiglass panel as they walked across the CatWalk platform to their home-cage on the other end. Balance support was provided in one of two ways: by the experimenter holding the tail, or by narrowing the lateral walls of the CatWalk such that the rats remained upright. These video recordings were digitized with MaxTraq Pro and analyzed with MaxMate (Innovision Systems). The evaluation of treadmill locomotion, over-ground locomotion and footprint analysis were compared at 6 weeks post-injury, just before the terminal electrophysiological procedure.

### Treadmill evaluation

Paint markers were placed over forelimb and hindlimb joints as previously discussed. Assessments were performed in one of two ways: quadrupedal stepping to examine interlimb coordination, and hindlimb-only locomotion, where the forelimbs were placed on a stationary platform 3 mm above the treadmill belt and only hindlimb stepping was evaluated. The experimenter provided balance support by holding the tail.

The recovery of treadmill locomotion was evaluated at 2, 4 and 6 weeks post-injury at treadmill speeds of 7.4 and 15 cm/s; locomotor performance for 10 steps at 15 cm/s is reported. The rats were tested with and without perineal stimulation (squeezing the base of the tail). Perineal stimulation was included in the testing paradigm, and applied in a manner that is consistent with other groups (Barbeau *et al.*, 1993; Bennett *et al.*, 1996; Boyce *et al.*, 2007; Côté*et al.*, 2011), in order to fully assess the reorganization of the locomotor circuitry post-Tx and with the neurotrophic factor treatments. To avoid any training effect that might occur because of testing, rats were evaluated for only 60 s at each speed. Video footage was then digitized as above to generate kinematic measures of stepping behavior, such as step length and angular joint excursion. The number of plantar hindlimb steps executed during each evaluation session was also quantified (plantar index = number of plantar steps executed per second during the evaluation session). The plantar index was then used to normalize step length [plantar step length = mean (stride length × plantar index)].

### Electrophysiology

#### Preparation of animals

Rats were anesthetized with an intraperitoneal injection of a ketamine hydrochloride (80 mg/kg) and xylazine (10 mg/kg) solution supplemented as required by intrajugular infusion of a 20% mixture of the ketamine/xylazine solution in dextran lactated Ringer’s solution. End-tidal CO_2_ (25–30 mmHg) was monitored through a tracheal canula, which also provided access for emergency ventilation if necessary. Body temperature was maintained at 35–37 °C with a circulating water blanket.

The lumbar spinal cord was exposed by dorsal laminectomy, and the nerves to the left (or, in some cases, the right) medial gastrocnemius (MG) and lateral gastrocnemius–soleus (LGS) muscles were dissected free, cut, and placed on hook electrodes for stimulation. Microscissors were used to open the dura, which was then carefully pinned aside with minutein pins. The L4 and L5 roots were mounted on hook electrodes in continuity for recording of the afferent volley. Exposed tissues were covered with warm mineral oil maintained at 37 °C.

#### Intracellular recording

Glass electrodes (20–25-mΩ resistance) filled with 3 m potassium acetate were used for intracellular motoneuron recording. Motoneuron identity was established by the response to antidromic stimulation of the ipsilateral MG or LGS nerves at twice threshold. Measures of motoneuron rheobase (Rh) and input resistance (*R*_N_) were obtained by using 20-ms depolarizing pulses of increasing amplitude (Rh) or 1 nA (*R*_N_). Afterhyperpolarization (AHP) amplitude was measured following the action potential initiated by a brief depolarization of the motoneuron via the intracellular electrode. Five to 30 sweeps were averaged for each measure, and only cells with membrane potentials between −60 and −80 mV were kept for analysis. Excitatory postsynaptic potentials (EPSPs) were recorded from identified motoneurons in response to 0.5-Hz stimulation of the heteronymous nerve or the ventrolateral funiculus (VLF) rostral (T8) or caudal (T13/L1) to the lesion at twice threshold. Generally, the response to 20 stimuli was averaged. We recorded from 5 to 10 motoneurons in each preparation.

#### Histology

Immediately after the electrophysiological experiments, rats were euhtanized by urethane overdose. Following transcardial perfusion with 0.1 m phosphate-buffered saline and cold 4% paraformaldehyde, spinal cord tissue was immediately dissected, post-fixed for 1 h in 4% paraformaldehyde, and cryoprotected in 30% sucrose at 4 °C for 4–7 days. Frozen sections (20 μm) of these spinal cords were then cut in series on a cryostat at −20 °C. Transverse sections of the L2–L5 spinal cord were stained with the following antibodies: rabbit anti-GFP (Invitrogen; 1 : 1000), mouse anti-choline acetyltransferase (ChAT) (Millipore; 1 : 400), and rabbit anti-c-Fos (Abcam; 1 : 600). The lesion site was cut in horizontal section and double-stained for Nissl/myelin with 2% Cresyl Violet and Cyanine R to confirm the absence of spared myelinated fibers within the lesion (Boyce *et al.*, 2007; Ollivier-Lanvin *et al.*, 2009; Jin *et al.*, 2011).

For immunohistochemical staining, tissue sections were rinsed in Tris-buffered saline (TBS), blocked in a 2% goat serum/TBS solution with 0.1% Triton-X, and incubated in the primary antibody overnight at 4 °C. For visualization of the antibody, sections were again rinsed in TBS, and incubated in a fluorescent secondary antibody raised in goat and conjugated with Alexa Fluor 488 or Alexa Fluor 594 (1 : 400; Molecular Probes) for 2 h at room temperature. Sections were then rinsed and coverslipped with Fluoromount-G (Beckman Coulter) mounting medium.

Sections were imaged with confocal microscopy (Olympus). Optical sections were captured at 1-μm increments and flattened in the *z*-axis to give a two-dimensional image. Image-J software (Abramoff *et al.*, 2004) was then used to obtain the optical density and counts of c-Fos-positive nuclei in the dorsal (laminae I–V), intermediate (VI–VII) and ventral gray (VIII–IX) of the L2 and L4/L5 spinal cord.

### Statistical analysis

The post-Tx/pre-Tx hindlimb withdrawal latency ratio (Hargreaves) was compared between groups with a one-way repeated measures anova and the Student–Neuman–Keuls *post hoc* test. Plantar index was compared between the groups with a Kruskal–Wallis anova on ranks with Dunn’s *post hoc* test. Plantar step length was compared between the groups with a one-way repeated measures anova and the Tukey *post hoc* test. The Mann–Whitney rank sum test was used to compare plantar step length with and without perineal stimulation within groups. Rh, AHP amplitude and EPSP amplitude were compared between groups with a one-way anova and the Student–Neuman–Keuls *post hoc* test. The Kruskal–Wallis anova on ranks with Dunn’s *post hoc* test was used in cases with unequal variances. Significance for all tests was set at *P* < 0.05, and data are presented as mean ± standard error except where otherwise stated. Where possible, we compared averaged results from individual rats (*n* = number of rats). If this did not reveal a significant result, we evaluated the results in a less conservative manner, using averages across all cells as reported in the text. A two-way anova and the Tukey *post hoc* test were used to compare counts of c-Fos-immunoreactive nuclei between groups and within three regions: the dorsal, intermediate and ventral regions of the spinal gray. The Kruskal–Wallis anova on ranks with Dunn’s *post hoc* test was used to compare c-Fos expression between lumbar segments – L2 vs. L4/L5. SigmaPlot 11.0 for Windows (Systat Software, San Jose, CA, USA) was used for statistical analyses.

## Results

### Lesion completeness

**FIG. 1:**
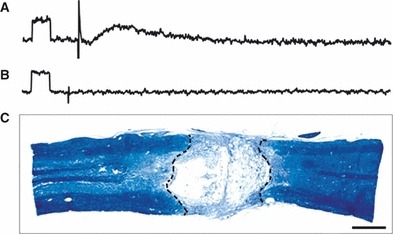
Verification of lesion completeness. Stimulation of the VLF below the lesion produced an EPSP in a recorded MG motoneuron (A). No response was observed with VLF stimulation above the lesion (B). Nissl/myelin staining of horizontal sections through the thoracic lesion site demonstrated the absence of spared myelinated fibers within the lesion cavity (C). Calibration pulse – 2 mV, 2 ms. Dashed lines indicate lesion edges. Scale bar – 500 μm.

During the surgical procedure, a separation of 0.5–1 mm was observed at the Tx site. Completeness of the spinal lesion was verified physiologically and histologically. Figure 1A shows that VLF stimulation (at T13) caudal to the lesion generated a synaptic response in a recorded MG motoneuron. Stimulation of VLF above the Tx (at T8) resulted in no response (Fig. 1B). This is consistent with the conclusion that no white matter was spared after Tx, as lateral white matter stimulation always produces a monosynaptic EPSP in ipsilateral lumbar motoneurons via intact fibers of the VLF (Petruska *et al.*, 2007; Hunanyan *et al.*, 2010). To further assess the completeness of the Tx injury, horizontal sections through the lesions of all of the rats were stained with Cresyl Violet/Cyanine R (Nissl/myelin). No myelinated fibers were seen (Fig. 1C). Together, these findings confirmed that all rats had completely transected spinal cords.

### GFP expression in the lumbar cord

**FIG. 2:**
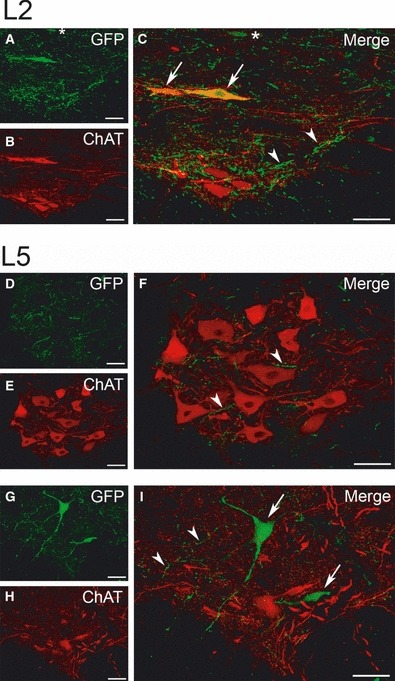
Localization of GFP (green) in L2 (A–C) and L5 (D–I) spinal cord of AAV–GFP-control rats. Motoneurons were stained with ChAT (red). In L2, GFP-positive motoneurons (C; arrows) and other ChAT-negative cells were observed (A–C, asterisk). Numerous GFP-positive fibers (C; arrowheads) were also present in L2. GFP-positive motoneurons were not observed in L5 in these animals (D–F); however, GFP-positive fibers (F–I; arrowheads) and cells (I; arrows) were present in this spinal segment. Scale bar – 50 μm.

We studied the transduction efficiency of AAV5 and the distribution of the transduced cells and their terminals by using GFP histochemistry in the AAV5–GFP-treated rats (*n* = 5). In order to obtain an evaluation of direct viral spread, we evaluated the expression of GFP in ChAT-labeled motoneurons, as these cells have processes limited to the vicinity of the cell body. We found GFP expression (Fig. 2A) in a few ChAT-positive motoneurons at L2 (Fig. 2B and C; arrows). In L5, no motoneurons were co-labeled with GFP (Fig. 2E and F, and H and I). These findings suggest limited transport of the virus far from the site of administration (T10). We also observed profuse GFP staining of fibers and varicosities at both L2 and L5 (arrowheads in Fig. 2). These were likely the result of infection of interneurons close to T10 with projections in the lumbar cord (Blits *et al.*, 2003). A few GFP-positive interneurons (ChAT-negative) were also observed (Fig. 2G and I, arrowheads); these were likely interneurons retrogradely labeled from axons projecting to the injury site. These findings suggest that most of the gene product associated with AAV reached the lumbar cord by intra-axonal transport of the gene product rather than by extracellular transport of the virus itself.

### Hargreaves test

**FIG. 3:**
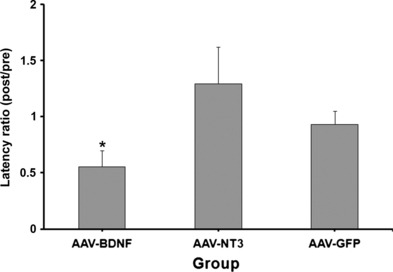
Ratio of post-Tx and pre-Tx thermal nociceptive threshold measured in the same rats with the Hargreaves test. Note the significant reduction in hindlimb withdrawal latency with AAV–BDNF treatment post-Tx. AAV–NT-3 and AAV–GFP did not influence thermal threshold.

Release of BDNF into the lumbar cord would be expected to sensitize the nociceptive pathway in the dorsal horn (Kerr *et al.*, 1999; Garraway *et al.*, 2003). This was examined by using the Hargreaves test, where rats in the three different treatment groups were examined pre-Tx and at 6 weeks post-Tx. One-way repeated measures anova indicated a significant reduction in hindlimb withdrawal latency post-Tx between the groups (*F*_2,8_ = 6.10, *P* = 0.025). Pairwise Student–Neuman–Keuls *post hoc* comparisons showed that AAV–BDNF treatment significantly reduced the latency of hindlimb withdrawal in response to noxious thermal stimulation, indicating sensitization post-Tx, as compared with AAV–NT-3-treated (*P* = 0.03) or AAV–GFP control (*P* = 0.04) rats (Fig. 3). Administration of AAV–NT-3 or AAV–GFP elicited no change in the latency of the response to the thermal stimulus.

### Hindlimb locomotor recovery and footprint analysis

**FIG. 4:**
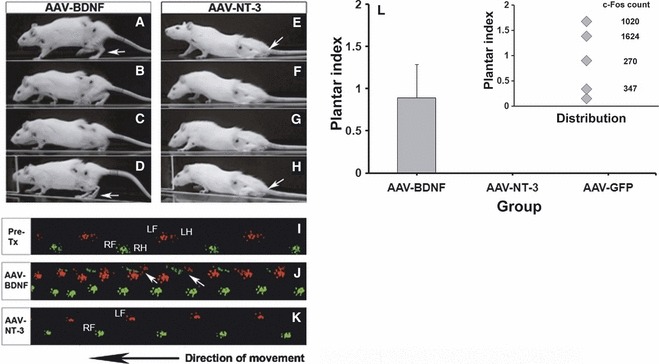
Over-ground stepping in adult spinally transected rats was promoted by AAV–BDNF but not by AAV–NT-3. Video stills of locomotor behavior are shown for one step (A–C) and as the rat exited the walkway (D) for AAV–BDNF-treated and AAV–NT-3-treated (E–G; H) rats. AAV–BDNF-treated rats were capable of over-ground, weight-supported plantar stepping (arrows in A and D). In contrast, AAV–NT-3-treated rats dragged their hindlimbs (arrows in E and H) as they progressed along the walkway. Footprint analysis indicated an alternating pattern of forelimb (RF and LF) and hindlimb (RH and LH) placement pre-Tx (I). In AAV–BDNF-treated rats (J), hindpaw use returned (arrow), although the animal’s torso was slightly twisted to the left, and forelimb–hindlimb coordination was absent. Only forelimb use was observed in AAV–NT-3-treated rats (K). The plantar indexes for the different treatments (inset, L) demonstrate that AAV–GFP-control and AAV–NT-3-treated rats could not plantar step (plantar index = 0), whereas all AAV–BDNF-treated rats had non-zero plantar step indexes. The number of c-Fos-positive nuclei is also shown where available (inset, L). RF, right forelimb; LF, left forelimb; RH, right hindlimb; LH, left hindlimb.

AAV–BDNF-treated rats could execute plantar weight-bearing hindlimb steps when tested on a stationary walkway. This over-ground stepping required the balance support provided by the plexiglass enclosure (Fig. 4A–D) or gently holding the tail. Although the AAV–BDNF-treated rats moved efficiently across the walkway using both forelimbs and hindlimbs, there were incidences of synchronous hindlimb activity resulting in hopping rather than alternating hindlimb stepping indicative of abnormal stepping behavior (see Video S1). In addition, footprint analysis with the CatWalk demonstrated that the hindlimbs were not coordinated with the forelimbs post-Tx (Fig. 4J, arrows) when compared with pre-Tx stepping, where there was a regular alternating forelimb–hindlimb stepping pattern (Fig. 4I).

Rats treated with AAV–NT-3 (Fig. 4E–H) or AAV-GFP controls (not shown) were incapable of plantar stepping over-ground despite the provision of balance support, and instead used only their forelimbs to pull themselves along the walkway (Fig. 4K). Quantification of the number of hindlimb plantar steps generated by each group of rats demonstrated that AAV–BDNF-treated rats generated significantly more plantar steps (Kruskal–Wallis one-way anova on ranks, Dunn’s *post hoc* test; *P* = 0.001) than rats in the other two groups (plantar index = 0) (Fig. 4L). The inset represents the plantar index of each AAV–BDNF-treated rat, and indicates some inter-animal variability within the group. However, each AAV–BDNF-treated rat had some degree of plantar stepping. No rats in the other two groups generated any plantar steps. Thus, AAV–BDNF, but not AAV–NT-3, treatment restored the capacity for plantar weight-supported, hindlimb stepping. We also noted that, by 4 weeks post-Tx, AAV–BDNF-treated (but not AAV–NT-3-treated) rats developed spontaneous hindlimb movements suggestive of spasticity.

### Treadmill locomotor recovery

**FIG. 5:**
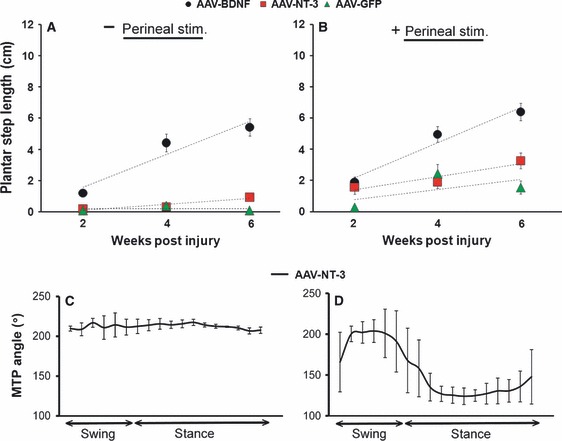
(A) Hindlimb kinematics for 10 steps during treadmill locomotion at 15 cm/s in spinally transected rats (6 weeks post-Tx). Without perineal stimulation, (A) AAV–BDNF-treated rats took significantly longer hindlimb plantar steps than AAV–NT-3-treated or AAV–GFP-control rats. With perineal stimulation (B), plantar step length significantly improved for AAV–NT-3-treated rats at all three time points, and transiently improved for AAV–GFP-treated rats. The range of motion at the MTP joint in a representative AAV–NT-3-treated rat shows the inability to plantar step in the absence of perineal stimulation (C). However, with perineal stimulation, flexion about the MTP joint returned (D) and rats were capable of plantar stepping. (Mean ± standard deviation for 10 steps).

Recovery of treadmill locomotion was evaluated every 2 weeks with and without perineal stimulation. In the absence of such stimulation, AAV–NT-3-treated and AAV–GFP-control rats were incapable of plantar hindlimb stepping at any time point (Fig. 5A). One-way repeated measures anova indicated a significant effect of treatment on plantar step length at 6 weeks post-Tx (*F*_2,94_ = 46.38, *P* < 0.001). Pairwise multiple comparisons (Tukey *post hoc* test) further demonstrated that rats receiving AAV–BDNF treatment generated significantly longer plantar weight-bearing hindlimb steps at the first evaluation session (2 weeks post-Tx) and for the duration of the study (up to 6 weeks) (Fig. 5A) than AAV-NT-3-treated or AAV–GFP-control rats (*P* < 0.001).

The Mann–Whitney rank sum test indicated a significant improvement in plantar step length with perineal stimulation, as compared with plantar step length in the absence of this stimulation (*P* = 0.012), in AAV–NT-3-treated rats (Fig. 5B). One rat in the control group (AAV–GFP) displayed a small transient improvement in stepping performance at 4 weeks post-Tx, a phenomenon that has been described in other animal models of complete SCI (de Leon *et al.*, 1998b). Stepping by AAV–BDNF-treated rats was not greatly improved by perineal stimulation. The improvement in stepping elicited by perineal stimulation in AAV–NT-3-treated rats is further demonstrated by examination of MTP joint excursion during stepping. In the absence of perineal stimulation, AAV–NT-3-treated rats stepped on the dorsum of the hindpaw, with hyper-extension of the MTP joint throughout the step cycle (Fig. 5C). However, while perineal stimulation was applied (Fig. 5D), dorsiflexion and greater excursion were achieved at the MTP joint, resulting in significantly improved hindlimb stepping performance (Fig. 5D).

### Motoneuron physiology

**FIG. 6:**
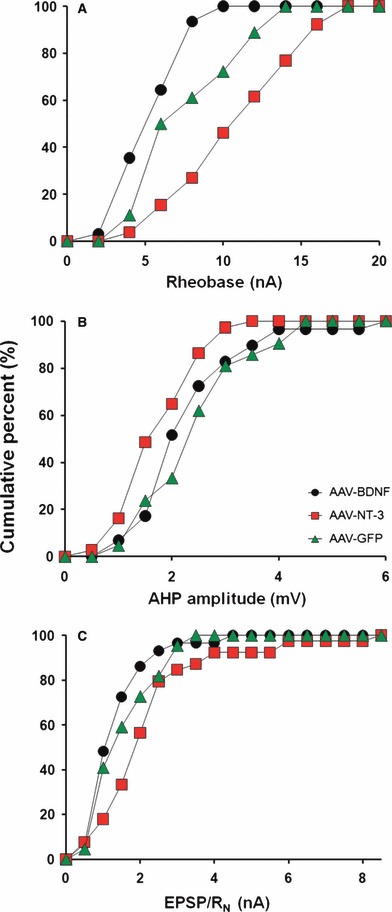
Cumulative frequency distributions of Rh (A), AHP amplitude (B), and normalized monosynaptic EPSP amplitude (C). The leftward shift in the distribution indicates reduced Rh in motoneurons of AAV–BDNF-treated rats as compared with AAV–GFP-treated rats. The rightward shift indicates the opposite effect on motoneuron Rh of AAV–NT-3 (A). AHP amplitude decreased (leftward shift) in AAV–NT-3-treated rats (b). A small increase in normalized EPSP amplitude (rightward shift) was observed in AAV–NT-3-treated rats (C).

We observed that treatment with AAV–BDNF or AAV–NT-3 had different effects on the physiology of ankle extensor motoneurons measured at 6 weeks post-Tx (Fig. 6A). In AAV–GFP-control rats, the average (mean ± standard deviation) Rh of MG and LGS motoneurons was 7.9 ± 2.1 nA (*n* = 5 rats), similar to the mean value obtained in untreated adult rats spinally transected at T8 (7.3 ± 4.0 nA) (Button *et al.*, 2008). One-way anova was used to compare Rh between the groups (*F*_2,13_ = 17.06, *P* < 0.001). Student–Newman–Keuls pairwise *post hoc* analysis indicated that, with AAV–NT-3 treatment, mean Rh significantly increased to 11.4 ± 1.9 nA (*n* = 6 rats; *P* = 0.005) as compared with AAV-GFP controls. In contrast, in rats treated with AAV–BDNF, Rh was significantly reduced to 5.4 ± 0.8 nA as compared with controls [*n* = 5 rats; *P* = 0.038 (no electrophysiological data were obtained from one AAV–BDNF-treated animal)]. Thus, it is apparent that, at the viral titers used, BDNF increases the excitability of ankle extensor motoneurons (lower Rh), whereas NT-3 decreases it (higher Rh).

Other variables measured in these motoneurons were affected by NT-3 but not by BDNF. In AAV–GFP-control rats, AHP amplitude averaged 2.4 ± 1.0 mV (21 cells), similar to the mean value (2.6 ± 1.5 mV) in untreated control transected adult rats (Button *et al.*, 2008). Kruskal–Wallis one-way anova on ranks was used to compare AHP amplitude between groups. Dunn’s pairwise *post hoc* test indicated that, after AAV–NT-3 treatment, the mean value of AHP amplitude fell to 1.7 ± 0.7 mV (39 cells) (Fig. 6B), significantly lower than that in AAV–GFP-control rats (*P* = 0.007). In contrast, the mean AHP amplitude in AAV–BDNF-treated rats did not differ from that of controls, at 2.2 ± 1.0 mV (39 cells). Similarly, NT-3, but not BDNF, affected the strength of the monosynaptic projection to motoneurons. Because we also found that NT-3 reduced motoneuron input resistance, whereas BDNF had no measurable effect, we normalized EPSP amplitude in each motoneuron to take account of differences in input resistance by expressing it in units of synaptic current (EPSP/*R*_N_). Kruskal–Wallis one-way anova on ranks with Dunn’s *post hoc* test indicated that EPSP/*R*_N_ was significantly greater in AAV–NT-3-treated rats (2.2 ± 1.6 nA, 39 cells; *P* = 0.007) than in AAV–BDNF-treated rats (1.3 ± 0.9 nA, 29 cells) (Fig. 6C). There was no significant difference in EPSP/*R*_N_ between the AAV–BDNF group and the AAV–GFP group (1.5 ± 0.9 nA, 22 cells) (*P* > 0.05). We conclude that BDNF and NT-3 exert different physiological effects on ankle extensor motoneurons and their synapses.

### Site of neurotrophin action

We have reported different electrophysiological changes in ankle extensor motoneurons recorded in L4/L5 in response to AAV–BDNF and AAV–NT-3. As the inputs to the central pattern generator (CPG) for locomotion in the rat are in the upper (L1/L2) lumbar spinal segments (Cazalets *et al.*, 1995), we used c-Fos immunohistochemistry to investigate the effects of neurotrophin treatment on cells located in the vicinity of the CPG in the same rats as those studied electrophysiologically. Although *c-Fos* expression is generally used to identify spinal neurons activated by nociceptive stimuli (Hunt *et al.*, 1987) or during stepping behavior (Ichiyama *et al.*, 2008), expression of this immediate early gene is also directly affected by neurotrophins (Morgan & Curran, 1986; Bartel *et al.*, 1989; Sheng *et al.*, 1990), and has been used to identify spinal neurons activated by neurotrophins (Kerr *et al.*, 1999). Thus, in these experiments, the spinal cords of AAV–BDNF-treated or AAV–NT-3-treated rats were evaluated for c-Fos expression in order to visualize neuronal populations activated because of chronic neurotrophin release. AAV–GFP preparations were used as a control.

**FIG. 7:**
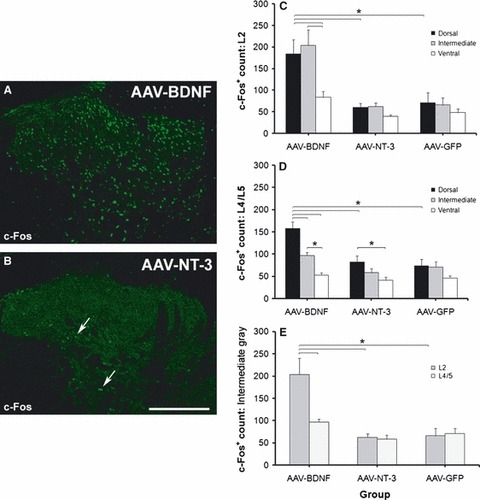
Comparison of c-Fos immunoreactivty in L2 and L4/L5. Rats treated with AAV–BDNF (A) showed greater numbers of c-Fos-positive nuclei than AAV–NT-3-treated (B) or AAV–GFP-control rats. These nuclei were concentrated in the dorsal and intermediate laminae of L2 (C) and L4/L5 (D). Significantly greater numbers of c-Fos-positive nuclei were present in the intermediate gray in L2 than in L4 (E). * indicates significant differences between groups. Further statistical details are given in the text. Scale bar – 200 μm.

Overall, the number of c-Fos-positive nuclei was greater in the lumbar cords of AAV–BDNF-treated rats than in those of AAV–NT-3-treated rats (compare the examples in Fig. 7A and B). Two-way anova demonstrated a significant effect of dorsoventral region (*F*_2,129_ = 6.57, *P* = 0.002) and of treatment (*F*_2,129_ = 20.91, *P* < 0.001) on c-Fos staining density in the lumbar cord. Evaluation of c-Fos immunostaining within three dorsoventral regions of the L2 spinal gray with the Tukey *post hoc* test showed significantly elevated numbers of c-Fos-positive nuclei in the dorsal and intermediate gray of AAV–BDNF-treated rats (*P* < 0.001) as compared with the same regions in rats treated with AAV–NT-3 or AAV–GFP (Fig. 7C). The number of c-Fos-positive nuclei was also elevated in the L4/L5 dorsal horn (*P* < 0.001) after AAV–BDNF treatment as compared with AAV–NT-3-treated or AAV–GFP control rats (Fig. 7D and E). These results are consistent with the enhanced stepping performance of AAV–BDNF-treated rats, as this is a function of cells in the intermediate gray in L2 (see Discussion). They are also consistent with the increase in sensitivity to nociceptive stimuli delivered to the footpad observed in the AAV–BDNF-treated rats, as the footpad is in the L4/L5 dermatomes (Takahashi & Nakajima, 1996) (Fig. 7D).

## Discussion

Here, we have compared the effects of BDNF or NT-3 on the extent and mechanisms of locomotor recovery in adult rats spinally transected at T10. These neurotrophins were administered at the Tx site by AAV5s engineered with the gene for BDNF or NT-3. The immunohistochemical results obtained with AAV–GFP suggest that interneurons near the injection site were infected (Blits *et al.*, 2003) and that their axons transported the gene product (Zhou & Rush, 1996; Altar & DiStefano, 1998; Coumans *et al.*, 2001) to the lumbar cord. There is ample evidence that both BDNF and NT-3 can be secreted from cells to affect surrounding and distant cells once transported (Lim *et al.*, 2003; Kuczewski *et al.*, 2009). As many fibers and varicosities were labeled with GFP in L2 and L5, it seems likely that infection with AAV–BDNF or AAV–NT-3 resulted in the release of these neurotrophins onto cells of the CPG in L2 (Jakeman *et al.*, 1998; Blits *et al.*, 2003) and motoneurons in L5, which are known to express both TrkB and TrkC receptors (Yan *et al.*, 1997; Copray & Kernell, 2000), whose excitability was tested directly.

The behavioral, electrophysiological and immunohistochemical results suggest that chronic BDNF expression raised the excitability of spinal cord neurons, which improved hindlimb locomotion but had the detrimental effect of increasing spasticity and sensitizing nociceptive circuits. The c-Fos expression studies indicating BDNF-mediated activation of cells in the superficial dorsal horn where nociceptive inputs are processed support this finding. In addition, the reduced threshold of motoneurons, measured directly in AAV–BDNF-treated rats, undoubtedly contributed to spasticity. In contrast, NT-3 expression reduced cellular excitability, did not alter nociceptive sensitivity or elicit hindlimb spasticity, and generated only moderate changes in locomotion that required treadmill assistance and extrinsic stimulation. Administration of AAV–GFP resulted in no behavioral or excitability changes, but demonstrated the ability of AAV to infect spinal neurons. The same viral titer was used in all experiments; thus, we believe that the different results were caused by the different viral products.

The completeness of the Tx was verified by direct visual inspection during the surgical procedure, as well as by electrophysiological and histological tests. Furthermore, placing Gelfoam in the cavity prevented apposition of the severed ends of the cord. We did not observe myelinated fibers crossing the lesion in rats from any of the three groups. Neurotrophin treatment does result in axonal sprouting in different SCI models, and has been observed by other groups (Schnell *et al.*, 1994; Blits *et al.*, 2003; Fortun *et al.*, 2009; Bonner *et al.*, 2011). Thus, although the lesions were verified as being complete, we cannot rule out the possibility that local sprouting could have contributed to the locomotor recovery observed in these groups of rats.

The concept that isolated lumbar segments can mediate hindlimb stepping in the absence of descending input from higher brain centers has a long history, beginning with the discovery of the CPG in lower vertebrates (Grillner, 1974, 1985; Harris-Warrick & Cohen, 1985) and the ability of locomotor training to restore stepping in adult spinally transected cats (Lovely *et al.*, 1986; Barbeau & Rossignol, 1987). More recent studies in mammals have indicated that adult rats spinally transected as neonates can be bipedally treadmill-trained to step with external weight support (Timoszyk *et al.*, 2005; Ichiyama *et al.*, 2008). Rats spinally transected as adults can step if training is supplemented with epidural stimulation (Ichiyama *et al.*, 2005; Courtine *et al.*, 2009).

The present work further advances our understanding of the restoration of stepping mediated by isolated spinal segments in two major ways. First, it demonstrates that the individual neurotrophic factors BDNF and NT-3, chronically expressed via an AAV construct delivered at the time of injury, can elicit long-lasting recovery of locomotion. Interestingly, these neurotrophins have disparate effects on the recovery of locomotion following complete SCI in the adult rat. Specifically, BDNF is effective, without training, in activating locomotor circuitry in the rodent lumbar cord (see also Jakeman *et al.*, 1998; Blits *et al.*, 2003) to promote over-ground plantar locomotion as well as treadmill-assisted stepping in the adult mammal following a complete spinal lesion. In contrast NT-3’s effect is limited to promoting treadmill-assisted locomotion requiring concomitant perineal stimulation. That BDNF expression at the site of injury in the spinal cord could elicit sustained hindlimb locomotor recovery is an important advance in identifying molecules to direct spinal plasticity towards a beneficial outcome post-SCI.

A second important conclusion is that BDNF enhances motoneuron excitability and, by inference, the excitability of TrkB-expressing interneurons in the spinal cord, thus providing a likely mechanism by which BDNF promotes stepping after SCI. Although we cannot identify the class(es) of interneurons that have been activated, their location in the intermediate zone of L2, as revealed by c-Fos immunohistochemistry, makes it likely that cells in the CPG required for stepping have been activated (Jankowska & Roberts, 1972; Burke *et al.*, 2001; Jankowska, 2001; Grillner, 2002; Jankowska & Hammar, 2002; Kiehn *et al.*, 2010). In contrast, NT-3 reduces motoneuron excitability and does not substantially enhance c-Fos expression or elicit spontaneous over-ground stepping. However, NT-3 causes other electrophysiological changes that may facilitate treadmill-assisted stepping (see below).

The recovery of stepping elicited by exogenous BDNF and NT-3 after SCI is consistent with previous findings that locomotor recovery after step training of spinally transected rats is associated with elevation of spinal cord levels of BDNF, NT-3, and neurotrophin 4 (Gomez-Pinilla *et al.*, 2001; Skup *et al.*, 2002; Côté*et al.*, 2011). Furthermore, Boyce *et al.* (2007) demonstrated that treadmill-assisted hindlimb stepping in adult spinally transected cats could be restored by exogenous neurotrophic factor administration in the absence of step training by using a mixture of BDNF-expressing and NT-3-expressing fibroblasts delivered at the lesion site. However, over-ground stepping was not tested in these studies, and perineal stimulation was used to varying extents during evaluation sessions. The same group reported the ability of either BDNF-expressing or NT-3-expressing fibroblasts to promote treadmill-assisted hindlimb stepping in spinally transected cats while using perineal stimulation (Ollivier-Lanvin *et al.*, 2008). Delivery of AAV–BDNF to L2 of adult spinally transected rats also results in treadmill-assisted stepping (Ziemlinska *et al.*, 2010).

The excitability changes in motoneurons treated with BDNF or NT-3 are consistent with those previously observed with these neurotrophins under different conditions. In normal rats, intramuscular BDNF treatment reduced motoneuron Rh (Gonzalez & Collins, 1997), whereas an increase was reported after intramuscular NT-3 treatment (Petruska *et al.*, 2010). These disparate effects of BDNF and NT-3 on motoneuron excitability, both presumably caused by paracrine or autocrine actions in the spinal cord, may result from differences in the ion channels that they affect. In SK-N-SH human neuroblastoma cell cultures (Lesser *et al.*, 1997), BDNF application increased sodium and calcium channel expression, which would increase excitability, whereas NT-3 increased potassium channel expression, which would decrease it. Therefore, each neurotrophic factor has its own electrophysiological signature, which may be used to predict the changes in cell excitability and perhaps recovery of plantar stepping when it is expressed in the spinal cord post-SCI.

The number of c-Fos-positive nuclei in AAV–BDNF-treated rats was greater in L2 than in L4/L5. This is consistent with earlier findings, where bipedally step-trained adult spinally transected rats showed greater numbers of c-Fos-positive nuclei in upper (L2) than in lower (L4/L5) lumbar spinal segments (Ichiyama *et al.*, 2008). Ichiyama and colleagues also noted the pruning of active interneuronal pools with treadmill training. It is difficult to directly compare c-Fos expression in these two experiments, because the step-trained rats were also subjected to 1 h of treadmill stepping before the animals were euthanized. In contrast, the BDNF-treated rats were not stepped, but rather were anesthetized during the electrophysiological experiment and killed upon its completion. It would be of interest to compare the roles of these two forms of activity (step training and prolonged treadmill stepping prior to sacrifice) on c-Fos expression in AAV–BDNF-treated rats.

A number of reports have demonstrated an increase in muscle spindle (group Ia) fiber-evoked EPSPs in motoneurons after NT-3 treatment (Arvanian *et al.*, 2003; Petruska *et al.*, 2010). Here, we did not observe an increase in the raw EPSP amplitude, but this was probably because of the concomitant decrease in input resistance of the motoneurons, which was also reported after treatment with NT-3 (Petruska *et al.*, 2010). When EPSP amplitude was normalized (by dividing by motoneuron input resistance), treatment with AAV–NT-3 was revealed to have enhanced the synaptic action of group Ia fibers. Both the decrease in motoneuron AHP amplitude and the increase in synaptic action of group Ia fibers may have contributed to the treadmill stepping observed in NT-3-treated spinally transected rats by increasing motoneuron discharge frequency in response to the proprioceptive activity provided by the treadmill. Our results support the importance of the stretch reflex in stepping, which has been demonstrated previously (Pearson *et al.*, 2003; Yakovenko *et al.*, 2004).

Perineal stimulation was required for successful plantar stepping by AAV–NT-3-treated rats. Although we did not quantify the magnitude of this stimulus, it was delivered in a manner consistent with that reported by others (Barbeau *et al.*, 1993; Bennett *et al.*, 1996; Boyce *et al.*, 2007; Côté*et al.*, 2011). BDNF might enhance excitability directly, whereas constant perineal stimulation would add tonic synaptic drive to that of the synaptic input derived from treadmill-activated cutaneous, joint and muscle receptors in the hindlimb. Thus, the combination of these synaptic inputs was sufficient to counteract the NT-3-induced decrease in excitability to permit treadmill-assisted stepping. Epidural stimulation in earlier studies has been shown to facilitate stepping, presumably also by enhancing spinal cord excitability (Courtine *et al.*, 2009).

Biomechanical differences between over-ground and treadmill-assisted stepping may also be very important for the interpretation of these results. During treadmill locomotion, the hindlimbs are moved passively through the stance phase of the step cycle by the treadmill belt. During over-ground locomotion, successful stepping requires the rat to load the hindlimb through stance and to initiate the swing phase of the step cycle. Biomechanically, hip extension is critical to the initiation of stepping in the cat (McVea *et al.*, 2005). As AAV–BDNF-treated rats are able to support their weight and step independently over-ground, it will be important to confirm whether hip extensor motoneurons are also made more excitable by AAV–BDNF.

In summary, the present work provides behavioral evidence and a physiological rationale for further examination of the role of neurotrophins, and BDNF in particular, in locomotion. The demonstration that BDNF can lead to a long-lasting facilitation of stepping movements could potentially have important translational implications. However, a number of problems need to be addressed before it can be considered for more extensive clinical testing. As we report here, chronic BDNF expression with the use of AAVs resulted in increased sensitivity to noxious heat stimulation as well as spasticity, both of which detract from the clinical applicability of this treatment to promote locomotion after spinal injury with current viral delivery methods and constructs. Solutions to this problem will require genetic tools to provide strict temporal and spatial control of virally mediated BDNF expression. Finally, the present experiments were conducted in transected preparations to demonstrate the capacity of the fully isolated spinal cord. A further question is whether this approach will be equally beneficial in the contused cord, where some white matter conduction remains and can be improved (Hunanyan *et al.*, 2010).

## Abbreviations

AAV: adeno-associated virus
AHP: afterhyperpolarization
BDNF: brain-derived neurotrophic factor
CDS: coding sequence
ChAT: choline acetyltransferase
CPG: central pattern generator
EPSP: excitatory postsynaptic potential
GFP: green fluorescent protein
LGS: lateral gastrocnemius–soleus
MG: medial gastrocnemius
MTP: metatarsophalangeal
NT-3: neurotrophin 3
Rh: rheobase
*R*_N_: input resistance
SCI: spinal cord injury
TBS: Tris-buffered saline
Trk: tropomyosin-related kinase
Tx: transection
VLF: ventrolateral funiculus

## Supporting Information

**Video S1.** Video shows AAV-NT-3 and AAV-BDNF treated rats at 6 weeks post-Tx, crossing a stationary platform surrounded by a Plexiglas enclosure. The walls of the enclosure were narrowed to provide balance. AAV-NT-3 treated rats could not plantar step when tested in this manner. However, AAV-BDNF treated rats were capable of plantar weight supported hindlimb stepping.

Please note: As a service to our authors and readers, this journal provides supporting information supplied by the authors. Such materials are peer-reviewed and may be re-organized for online delivery, but are not copy-edited or typeset by Wiley-Blackwell. Technical support issues arising from supporting information (other than missing files) should be addressed to the authors.
